# Relationship between university students’ physical activity and mobile phone dependence: Mediating effect of subjective well-being and moderating effect of psychological capital

**DOI:** 10.3389/fpsyg.2022.983487

**Published:** 2022-12-29

**Authors:** Tianci Lu, Caixia Wang, Hanwen Chen, Baole Tao, Yueyan Jiang, Haoran Sui, Jun Yan

**Affiliations:** College of Physical Education, Yangzhou University, Yangzhou, Jiangsu, China

**Keywords:** physical activity, mobile phone dependence, subjective well-being, psychological capital, moderated mediation

## Abstract

**Background:**

With the ongoing development of the information society, the Internet and smartphones have become an essential way of life, but also fostered the problem of mobile phone dependence. Physical activity and subjective well-being have both been shown to correlate with mobile phone dependence, but the impact of subjective well-being on the relationship between physical activity and mobile phone dependence is not fully understood. This study investigates subjective well-being as a potential mediating variable in the relationship. It also investigates whether psychological capital moderates the association between subjective well-being and mobile phone dependence.

**Methods:**

A total of 9,569 students from 38 universities in Jiangsu Province were selected. Participants were surveyed *via* the online questionnaire distribution platform Questionnaire Star. Common method bias test and Pearson correlation tests were used to analyze the study indicators, and the theoretical model for this study was validated using Process plug-in developed by Hayes and set at *p* < 0.05 (two- tail) as statistically significant.

**Results:**

The levels of physical activity, subjective well-being, and psychological capital were all significantly higher for male students than female students. However, female students had a significantly higher level of mobile phone dependence. As predicted, there was a direct negative correlation between physical activity and mobile phone dependence, and subjective well-being mediated that relationship. Psychological capital moderated the relationship between subjective well-being and mobile phone dependence. It also positively moderated the indirect effect between physical activity and mobile phone dependence *via* subjective well-being.

## Introduction

In the information age, the rapid development of network technology and terminal devices has brought about revolutionary changes to human society and people’s lifestyles. According to the China Internet Network Information Center (2022), as of December 2021, China’s Internet penetration rate had reached 73% and almost all users (99.7%) accessed the Internet *via* smartphones. The Internet’s ubiquity has brought great convenience and benefits to young people’s lives, with studies showing that smartphones can support academic development, facilitate interpersonal communication, and provide leisure and entertainment (Lepp et al., 2014; Chen, 2015). However, problematic Internet usage is a growing challenge, with excessive use leading to a range of physical and mental health problems, such as disrupted work schedules, insomnia, poor concentration, anxiety, depression, and substance abuse (Sanchez-Martinez and Otero, 2009; Eyvazlou et al., 2016; Elhai et al., 2017, 2019; Lee et al., 2017). Studies show that teens use mobile phones more frequently than other groups and may be more likely to contribute to mobile phone dependency problems (Tony et al., 2002). Therefore, it is of urgent practical importance to better understand the mechanisms underlying mobile phone dependence and explore possible interventions.

It is well-documented that physical activity positively contributes to an individual’s physical and mental health (Watanabe et al., 2016; Okuyama et al., 2021). Physical activity has been defined as “any physical movement in which energy expenditure due to skeletal muscle contraction is higher than that at rest” (Yuede et al., 2018). Common examples include walking, jogging, and doing housework. Physical activity has already been identified as a potential therapeutic modality to address the negative effects of mobile phone overuse (Awick et al., 2017). Physical activity is an effective preventive measure to improve the health status of individuals (Garber et al., 2011). The idea has been widely recognized and validated in the academic community. In addition to the considerable physical health benefits, physical activity can also benefit an individual’s mental health. Therefore, we concluded that there was a negative relationship between physical activity and individuals’ mobile phone dependence behavior. Studies have shown that physically inactive students are more likely to develop mobile phone dependency problems than physically active students (Pereira et al., 2020). At the same time, students who prefer physical activity have the lowest overall scores on mobile phone dependence, the weakest on the dimensions related to mobile phone dependence, and the lowest detection rate of mobile phone dependence (Yang et al., 2020; Xiao, 2022). In summary, this study proposes that physical activity may also influence the source of mobile phone dependence and thus reduce it.

*H1*: Physical activity is negatively related to mobile phone dependence among university students.

An important aim of this study is to explore the mechanism underlying the relationship between physical activity and mobile phone dependence. Based on an extensive review of many relevant studies, it appears that subjective well-being could be a key mediating variable between physical activity and mobile phone dependence. The most widely accepted view of subjective well-being is that proposed by Diener et al. (1984) and Diener and Lucas (1999): an individual’s overall assessment of his or her quality of life and life satisfaction according to his or her own criteria. Diener and colleagues assert that subjective well-being has two components: (a) an affective component, mainly comprising positive and negative emotions; and (b) a cognitive component, assessing the extent to which one’s ideal expectations and state of life are being met. Rogers (1959) noted that when an individual’s actual life experience falls below their ideal, this can lead to negative emotional experiences, maladjustment, and other psychological problems, as well as an overall reduction in subjective well-being. This state of being at odds with reality also affects individuals’ use of the Internet. According to the mood enhancement hypothesis (Jennings et al., 1984), an individual’s emotional state determines the type and duration of Internet use. An individual experiencing imbalance between ideal and reality is more likely to seek satisfaction in the virtual online world; if the positive stimuli it provides consistently compensate for negative emotions associated with the ideal–reality gap, mobile phone dependence may result (Chiu, 2014; Ahn, 2016; Horwood and Anglim, 2019).

Numerous studies have shown that physical activity can increase subjective well-being by improving physical fitness, reducing stress, easing mental tension, and increasing life satisfaction (Olsson et al., 2014; An et al., 2020). However, the feeling of well-being that comes from physical activity is unique and cannot be sourced from elsewhere (Diener, 2000).

*H2*: Subjective well-being mediates the relationship between physical activity and mobile phone dependence.

Given the predicted negative relationship between subjective well-being and mobile phone dependence, it is important to consider under what conditions this favorable association may be strengthened. Psychological capital is a positive developmental state that one exhibits while growing up. It comprises four main dimensions: optimism, hope, self-efficacy, and resilience (Luthans et al., 2015). Previous studies have found that both self-efficacy and resilience in the psychological capital dimension are negatively related to individuals’ mobile phone dependence behavior (Chen et al., 2022; Ma et al., 2022; Xiao and Huang, 2022). Individuals with low psychological capital lack sufficient coping resources when facing difficulties and challenges, which can easily lead to failure and thus negatively affect the process of subjective well-being in relation to mobile phone dependence (Li et al., 2014). In contrast, individuals with higher real-life psychological capital can strengthen the inhibitory effect of subjective well-being on mobile phone dependence and act as a protective factor to help individuals alleviate their mobile phone dependence problems. Therefore, the present study hypothesized that psychological capital might strengthen the negative relationship between subjective well-being and mobile phone dependence.

*H3*: Psychological capital positively moderates the relationship between subjective well-being and mobile phone dependence.

**Figure 1:**
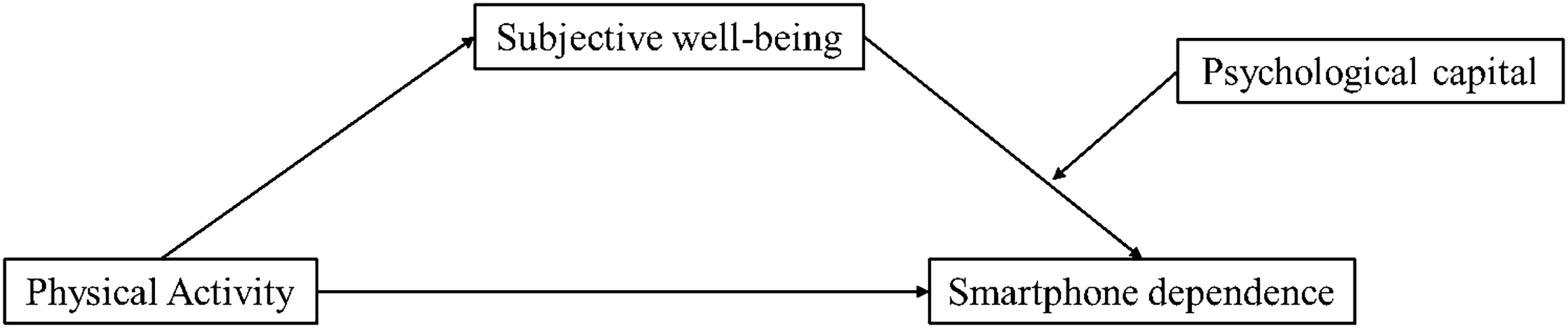
Theoretical model diagram.

*H4*: Psychological capital positively moderates the indirect effect between physical activity and mobile phone dependence *via* subjective well-being: the higher the level of psychological capital, the stronger the indirect effect (Figure 1).

Some studies point out that the different social and cultural roles of men and women, as well as clear gender labels for physical activity, result in women being less physically active than men (Lorber and Moore, 2007; Nielsen et al., 2011). Moreover, women show lower subjective well-being and psychological capital than men (Klein et al., 2003; Beauregard, 2012), but higher dependence on mobile phones (Beranuy et al., 2009). Therefore, this paper also tests gender differences for each variable, seeking insights on whether any such differences must be considered in physical-activity interventions targeting mobile phone dependence.

In summary, physical activity has some kind of shaping role with mobile phone dependence among university students. In previous studies, there has been no discussion on psychological capital as a protective factor to strengthen the negative relationship between subjective well-being and mobile phone dependence. And to strengthen the mediating effect of physical activity on mobile phone dependence *via* subjective well-being. This study attempted to explore the mechanism of physical activity on mobile phone dependence among college students in an extensive sample survey in Jiangsu province and investigate the role of subjective well-being and psychological capital.

## Materials and methods

### Ethical approval

The study was carried out in accordance with ethical standards, and was approved by the Ethical Committee of Yangzhou University Medical College (No. YXYLL-2022-110).

### Sample

Suitable universities were identified from the list of general institutions of higher learning on the official website of the Ministry of Education of the People’s Republic of China. The search targeted public and private schools ranked as Double First-Class or ordinary and located in Central Jiangsu, Southern Jiangsu, Northern Jiangsu, or Nanjing. Of the 78 colleges and universities meeting these criteria, 38 were selected for inclusion in the study. An online questionnaire was distributed to students through the Questionnaire Star platform. Participants at each institution were selected using random sampling and self-reported their responses to all questionnaire items (Class teachers share the QR code of the questionnaire with students for completion). The survey was conducted from March to April 2022. A total of 10,863 questionnaires were returned; The questionnaires collected were considered invalid and deleted if there were no answers, missing answers, withdrawal from the questionnaire, checking multiple options for single choice questions, regular answers, or short response time. After excluding invalid responses, 9,569 valid questionnaires remained, representing an effective response rate of 88.1%.

### Research tools

#### International physical activity questionnaire-long form (IPAQ-LF)

The IPAQ-LF was originally compiled by the International Physical Activity Measurement Working Group in 2001, and subsequently translated into Chinese by Qu and Li (2004). The questionnaire comprises five sections: Occupation, Housework, Transportation, Leisure, and Recreation. Three intensity levels of PA were assessed and calculated through this questionnaire, including low intensity activity, moderate intensity activity, and high intensity activity. MET scores were calculated by multiplying the MET value for each activity by the duration (minutes) and frequency (days). Obtaining a weekly physical activity requires the sum of these three activities. The Cronbach’s α of the IPAQ-LF in this study was 0.81.

#### Mobile phone addiction index (MPAI)

The study employed Leung’s (2008) MPAI, with 17 questions across four dimensions: Out of Control, Withdrawal, Escape, and Inefficiency. Each item is answered on a five-point Likert scale, ranging from “never” (1) to “always” (5). Higher scores indicate higher levels of mobile phone dependency. Specific questions (3–6, 8–9, and 14–15) are used as screening items for mobile phone dependency. A participant responding “always” (5) or “often” (4) to five or more of these questions is considered to be mobile phone dependent. The Cronbach’s α of the MPAI in this study was 0.93.

#### General well-being scale

The General Well-Being Scale was compiled by Duan (1996). It comprises 18 questions across 6 dimensions: Concerns about Health; Energy; Satisfaction, and Interest in Life; Depressed or Happy Mood; Control over Emotions and Behavior; and Relaxation and Tension. Questions 1, 3, 4, and 8–14 are answered on a 6-point Likert scale, ranging from “extremely good” (1) to “very bad” (6); questions 2 and 5–7 use a 5-point Likert scale, ranging from “extreme” (1) to “none” (5); and questions 15–18 require responses on an 11-point Likert scale, ranging from “very negative” (0) to “very positive” (10). A higher overall score indicates a higher level of subjective well-being. The Cronbach’s α for the scale in this study was 0.8.

#### Positive psychological capital questionnaire

The Positive Psychological Capital Questionnaire (Zhang et al., 2010) includes 24 questions evenly distributed across 4 dimensions: Self-efficacy, Optimism, Hope, and Resilience. Each item is scored on a 7-point Likert scale, ranging from “no match at all” (1) to “full match” (7). Higher scores indicate greater positive psychological capital. The Cronbach’s α of the questionnaire in this study was 0.950.

### Data processing

The statistical software SPSS 26.0 was used to run exploratory factor analysis and Pearson’s correlation test to check for common method bias and analyze differences in the levels of each variable between male and female students, setting *p* < 0.05 (two-tail) as the required value to establish statistical significance. Next, correlations between the variables were explored to verify satisfaction of the conditions for testing mediating and moderating effects. Finally, tests of mediating effects (Model 4) and of mediating and moderating effects (Model 14) were conducted using the Process plug-in developed by Hayes (2013), including age, grade, gender, and ethnicity in the model as control variables.

## Results

### Demographic characteristics

Of the 9,569 participants whose data were analyzed (Table 1), 3,626 (37.9%) were male and 5,943 (62.1%) were female. The age range of participants was 16–29 years old, with the highest proportion (6,706, 70.1%) aged 19–20 years old. A total of 4,807 (50.2%) were freshmen and 4,401 (46.0%) were sophomores. Regarding university type, 345 (3.6%) were from first-class universities, 1,039 (10.9%) from top-discipline universities, 4,926 (51.5%) from provincial universities, and 3,259 (34.1%) from private universities. Finally, in terms of ethnicity, the vast majority of participants were Han (9,085, 94.9%), while the remaining 484 (5.1%) were from ethnic minorities.

**Table 1:** Demographic characteristics.

Variable	N (9,569)
Gender	
Male	3,626 (37.9%)
Female	5,943 (62.1%)
Age	
16–18	1,261 (13.2%)
19–20	6,706 (70.1%)
21–29	1,602 (16.7%)
Grade	
Freshman	4,807 (50.2%)
Sophomore	4,401 (46.0%)
Junior year	285 (3.0%)
Senior year	76 (0.8%)
School type	
First-class universities	345 (3.6%)
Top disciplines universities	1,039 (10.9%)
Provincial universities	4,926 (51.5%)
Private universities	3,259 (34.1%)
National	
Han nationality	9,085 (94.9%)
Ethnic minorities	484 (5.1%)

### Common method bias test

Harman’s one-factor test showed that 14 common factors had characteristic roots greater than 1. The first common factor explained 24% of the total variation, which is below the threshold value of 40%. It was thus inferred that there were no serious common method bias problems in this study.

### Descriptive analyses

As reported in Table 2, male students scored significantly higher than female students on physical activity (*t* = 17.52, *p* < 0.001), psychological capital (*t* = 5.01, *p* < 0.001), and subjective well-being (*t* = 7.00, *p* < 0.001). Male students also had a significantly lower level of mobile phone dependence compared to female students (*t* = −6.55, *p* < 0.001).

**Table 2:** Mean scores for males and females.

Variable	Male (*M* ± *SD*) (*n* = 3,626)	Female (*M* ± *SD*) (*n* = 5,943)	*t*	*p*	Cohen’s *d*
Physical activity	3,803.96 ± 3669.75	2,662.58 ± 2676.59	17.52	0.00^*^	0.355
Psychological capital	120.87 ± 22.88	118.68 ± 19.32	5.01	0.00^*^	0.11
Subjective well-being	79.26 ± 11.92	77.52 ± 11.72	7.00	0.00^*^	0.15
Smartphone dependence	41.86 ± 15.10	43.78 ± 13.12	−6.55	0.00^*^	−0.14

M, mean; SD, standard deviation. ^*^p < 0.01.

### Correlation analyses

Pearson’s correlation test was used to measure the correlations between physical activity, subjective well-being, psychological capital, and mobile phone dependence. As shown in Table 3, there was a significant negative correlation between physical activity and mobile phone dependence (*r* = −0.058, *p* < 0.001): this means that the higher the level of physical activity, the lower the level of mobile phone dependence. Physical activity was also significantly positively correlated with subjective well-being (*r* = 0.059, *p* < 0,001) and psychological capital (*r* = 0.119, *p* < 0.001). As also predicted, mobile phone dependence was significantly negatively correlated with psychological capital (*r* = −0.165, *p* < 0.001) and subjective well-being (*r* = −0.300, *p* < 0.001). Finally, subjective well-being was significantly positively correlated with psychological capital (*r* = 0.492, *p* < 0.001).

**Table 3:** Correlation analysis between variables.

Variable	*M*	*SD*	1	2	3	4
1. Physical activity	3,095.08	3,139.74	–	–	–	–
2. Psychological capital	119.51	20.77	0.119^*^	–	–	–
3. Subjective well-being	78.20	11.83	0.059^*^	0.492^*^	–	–
4. Smartphone dependence	43.05	13.93	−0.058^*^	−0.165^*^	−0.300^*^	–

M, mean; SD, standard deviation. ^*^p < 0.01.

### Mediating effects analysis

Based on the correlation analysis, mediating effects were analyzed using PROCESS Model 4 in SPSS (Hayes, 2013), setting physical activity as the independent variable, subjective well-being as the mediating variable, and mobile phone dependence as the dependent variable. As shown in Table 4, the overall regression equation was significant: *R^2^* = 0.0082, *F* = 15.7244, *p* < 0.01. A bootstrap method was then used to test the mediating effect for 5,000 samples. As Table 5 reports, the total effect of physical activity on mobile phone dependence was −0.0483 (Table 5).

**Table 4:** Mediation effects (standardized).

Variable	Subjective well-being			Smartphone dependence			Total effect		
	*β*	*SE*	*t*	*β*	*SE*	*t*	*β*	*SE*	*t*
Physical activity	0.0486	0.0103	4.7038^*^	−0.0325	0.0099	−3.2698^*^	−0.0483	0.0104	−4.6603^*^
Subjective well-being				−0.2914	0.0113	−25.9003^*^			
Control variable									
	*β*	*SE*	*t*	*β*	*SE*	*t*			
Gender	−0.1316	0.0214	−6.1368^*^	0.0806	0.0205	3.9232^*^			
Age	−0.0262	0.0118	−2.2161^*^	0.0114	0.0113	1.0129			
Grade	−0.0206	0.0212	−0.9702	0.0010	0.0203	0.0505			
National	−0.2140	0.0465	−4.6059^*^	0.0741	0.0445	1.6668			
*R^2^*	0.0110			0.0934			0.0082		
*F*	21.2163^*^			164.2336^*^			15.7244^*^		

SE, standard error. ^*^p < 0.01.

**Table 5:** Total, direct, and mediating effects.

Effect	Effect	Boot *SE*	Boot LLCI	Boot ULCI	Relative effect ratio
Total effect	−0.0483	0.0104	−0.0685	−0.0280	–
Direct effect	−0.0340	0.0099	−0.0534	−0.0145	70.4%
Mediating effect of subjective well-being	−0.0143	0.0031	−0.0205	−0.0083	29.6%

Boot SE, Bootstrap standard error; Boot LLCI, Bootstrap lower limit of the 95% confidence interval; Boot ULCI, Bootstrap upper limit of the 95% confidence interval.

Physical activity was a significant negative predictor of mobile phone dependence (*β* = −0.0325, *t* = −3.2698, *p* < 0.001), as was subjective well-being (*β* = −0.2914, *t* = −25.9003, *p* < 0.001). Moreover, physical activity was also significantly positively related to subjective well-being (*β* = 0.0486, *t* = 4.7038, *p* < 0.001). According to the bootstrap 95% confidence interval (CI), the direct effect of physical activity on mobile phone dependence was −0.0340 (95% CI [−0.0534, −0.0145]), with a relative effect of 70.4%. These values indicate a significant direct effect of physical activity on mobile phone dependence among participating university students. The mediating effect of subjective well-being was −0.0143 (95% CI [−0.0205, −0.0083]), with a relative effect of 29.6%, indicating a significant mediating effect. Therefore, H1 and H2 are both supported.

### Moderating effects analysis

**Figure 2:**
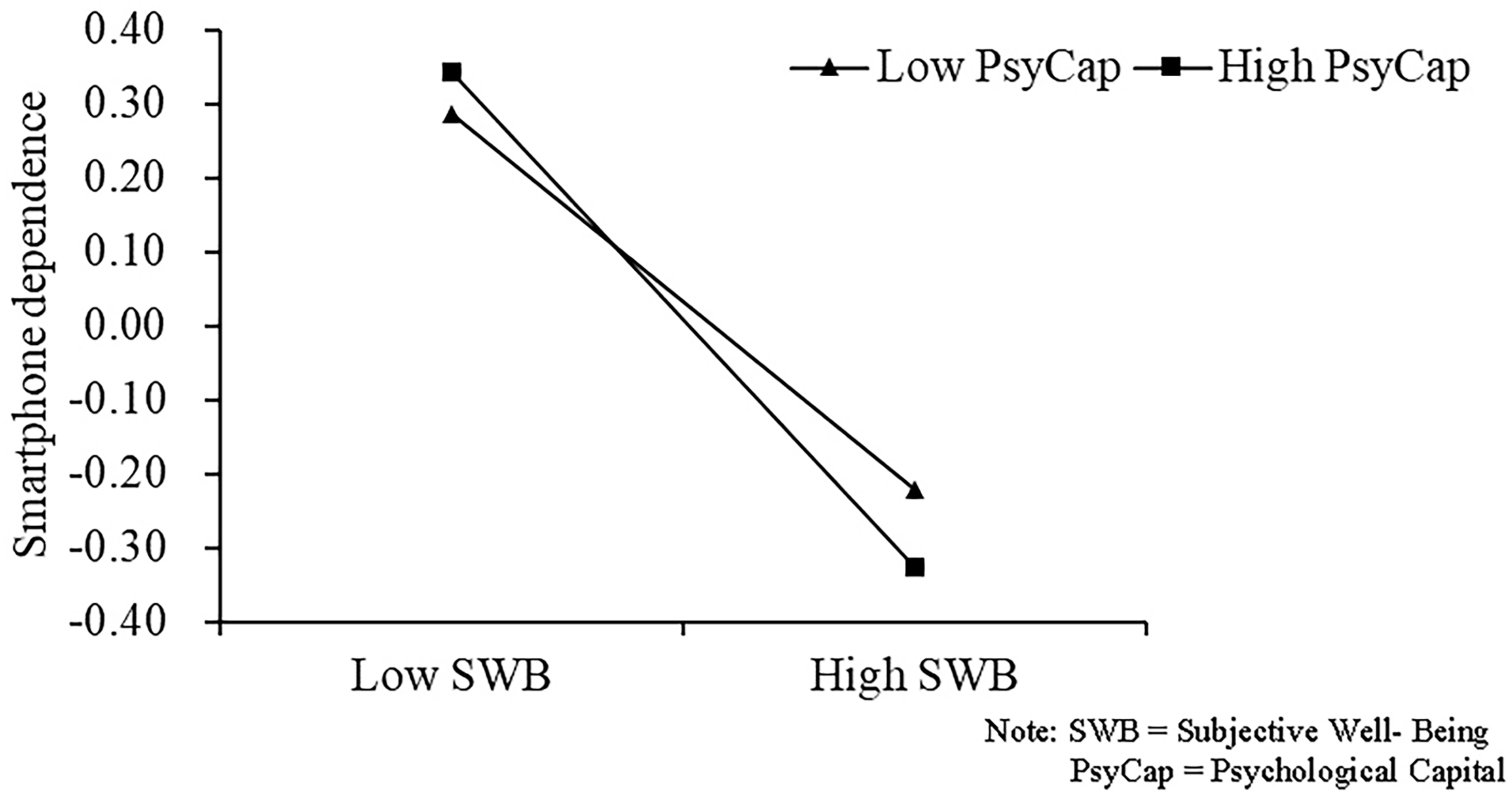
Simple slope plot.

The moderating effects were tested by including psychological capital as a moderating variable in PROCESS Model 14. Focusing first on the interaction term between psychological capital and subjective well-being, this was found to have a significant negative effect on mobile phone dependence (*β* = −0.0399, *p* < 0.001), indicating a moderating effect. Simple slope analysis showed that for university students with high psychological capital, subjective well-being had a significant negative association with mobile phone dependence (*β* = −0.3314, 95% CI [−0.3588, −0.3039]). For students with low psychological capital, the negative correlation between subjective well-being and mobile phone dependence was also significant (*β* = −0.2515, 95% CI [−0.2764, −0.2266]). As depicted in the simple slope plot (Figure 2), students with high (vs. low) psychological capital showed a significantly higher tendency to decrease their mobile phone dependence as their subjective well-being increased. These findings support H3. Regarding the final prediction, the indirect effect of physical activity on mobile phone dependence *via* subjective well-being was stronger in the high psychological capital group (*β* = −0.0161, 95% CI [−0.0234, −0.0088]) than in the low psychological capital group, and the difference between the indirect effects with high and low levels of psychological capital was significant [−0.0122 (95% CI [−0.0178, −0.0066]). Therefore, H4 is also supported.

## Discussion

In summary, the study found that, compared to their female peers, male university students had higher levels of physical activity, subjective well-being, and psychological capital but lower levels of mobile phone dependence. A direct negative relationship was identified between physical activity and mobile phone dependence. This association is mediated by subjective well-being, while psychological capital moderates the relationship between subjective well-being and mobile phone dependence.

### Gender differences in physical activity, subjective well-being, mobile phone dependence, and psychological capital

Studies around the world have shown that, on average, men engage in significantly higher levels of physical activity than women (Azevedo et al., 2007; Javier et al., 2018; Regina et al., 2018; Werneck et al., 2019). The findings of the current study are, thus, consistent with the literature.

In terms of subjective well-being, women are more likely to be depressed than men, and thus more vulnerable to negative emotions (Shmotkin, 1990; Diener, 2000; Klein et al., 2003). Globally, women’s average monthly earnings are 20% lower than those of men; this difference is 19% in China (International Labour Organization, 2018). There is also a significant gap in starting salaries between male and female university graduates (Qing and Zheng, 2013), which may contribute to women having significantly lower subjective well-being relative to men. This suggests that women’s lower subjective well-being may have complex social implications, and that schools should pay more attention to the subjective well-being and mental health education of female students.

Regarding psychological capital, this study’s results are consistent with numerous prior findings. In particular, several scholars have found that men have significantly higher resilience and self-efficacy compared to women (Bonanno, 2004; Beauregard, 2012) – both are key components of psychological capital.

As regards the finding that mobile phone dependence was significantly higher in female students, prior research indicates that female adolescents are more interested than males in building and maintaining social relationships, and consequently devote more time to interpersonal interactions and emotional engagement (Bianchi and Phillips, 2005; Beranuy et al., 2009). Female adolescents are also more inclined to use smartphones for interpersonal interactions and dealing with emotional problems (Beranuy et al., 2009). Thus, female adolescents use and check their mobile phones more frequently than do male adolescents, leading to the higher mobile phone dependence found in this study.

Given the identified differences between female and male university students, educators may need to devote more attention and effort to female students in physical-activity interventions to reduce mobile phone dependence.

### Physical activity and mobile phone dependence

The significantly negative correlation between physical activity and mobile phone dependence is consistent with prior findings. An increase in physical activity time reduces the amount of time spent on mobile phones, which can somewhat reduce dependence. Perhaps more importantly, physical activity can enhance enjoyment, reduce stress and tension, and bring subjective feelings of physical and mental pleasure (Awick et al., 2017). This could provide a viable alternative to use of gaming content, previously identified as the most influential factor in mobile phone dependence among university students (Abbasi et al., 2021). A 2013 study showed that physical activity has a suppressive effect on mobile phone addiction, mainly because the highly excited nerve cells are rested and adjusted after physical activity, which enhances the individual’s emotional adaptability to external changes, thus reducing the degree of mobile phone dependence (Kim, 2013). In addition, mobile phone dependence is often considered to have a strong association with an individual’s mental health (De Berardis et al., 2009), while physical activity can bring good psychological benefits to individuals (Hearing et al., 2016). Therefore, the negative correlation between physical activity and college students’ mobile phone dependence is predictable. Previous related studies also confirmed the findings of this paper (Zhong et al., 2021; Guo et al., 2022; Ren et al., 2022).

### Mediating role of subjective well-being

In finding that university students’ physical activity positively predicted their subjective well-being, this study supports the academic consensus that these variables are positively correlated (Garatachea et al., 2009; Buecker et al., 2021). Studies have shown that individuals feel positive emotions, including increased energy and vitality, within minutes of physical activity (Berger and Motl, 2000; Reed and Ones, 2006), as well as reductions in negative emotions such as fatigue and anxiety (Youngstedt, 2010; Johansson et al., 2011). Furthermore, individuals with high (vs. low) physical activity levels have significantly higher life satisfaction (An et al., 2020). The positive impact of physical activity on the emotional and cognitive levels of individuals is a component of subjective well-being. The experience of positive psychological feelings from physical activity encourages engagement in more physical activity, thus creating a positive cycle.

This study also found that subjective well-being negatively predicted mobile phone dependence, consistent with accumulating evidence of a negative relationship. According to theory on emotion enhancement, mobile phone dependence provides a form of psychological compensation for suffering (Jennings et al., 1984). Accordingly, as university students with lower subjective well-being tend to suffer more negative emotions and mental distress, they are susceptible to higher mobile phone dependence (Wang et al., 2021). Therefore, higher subjective well-being can effectively reduce mobile phone dependence, and serves to mediate the negative relationship between physical activity and mobile phone dependence among university students.

### Moderating effects of psychological capital

This study found that psychological capital moderates the mediating pathway between physical activity, subjective well-being, and mobile phone dependence. Relative to students with low levels of psychological capital, students with high psychological capital significantly strengthened the effect of subjective well-being in reducing mobile phone dependence and increased the indirect effect of physical activity on mobile phone dependence through subjective well-being. The I-PACE (interaction of person-affect-cognition-execution) model suggests that the interaction between individual susceptibility factors (genetic and personality), psychopathological factors (negative emotions) and cognitive and emotional factors leads to the development of interdependent behaviors in individuals (Brand et al., 2016). And psychological capital contains cognitive, motivational, decision-making, and emotional, which can influence individual susceptibility factors/psychopathological factors (negative emotions) that directly or indirectly impact the individual’s behavior (Caprara et al., 2008). Mobile phone dependence as an addictive behavior relates to the individual’s coping style. The lack of positive coping strategies and negative coping styles can lead to excessive use of mobile phones and the development of mobile phone dependence (Tang et al., 2014; Chen, 2015). In turn, the increased sense of the energy of psychological resources brought about by psychological capital enhances the development of cognitive abilities, influences motivation and decision-making, and protects the healthy development of individuals. Thus, individuals with high psychological capital can have positive cognitive and emotional experiences and reduce negative emotional influences (Youssef-Morgan and Luthans, 2015), thus reducing the risk of mobile phone addiction. It can be seen that psychological capital, as a positive emotional trait, can positively impact individuals’ psychological states. And when individuals experience positive emotions over time, their subjective well-being is better maintained and enhanced (Diener, 2012; Saltzman et al., 2020). Increased subjective well-being enables individuals to cope with addictive behaviors more effectively (Dong-Ling et al., 2010). In summary, psychological capital plays a moderating role in the mechanism of the negative relationship between subjective well-being and mobile phone dependence, increasing the positive effect of physical activity and subjective well-being on the influence of mobile phone dependence. University interventions targeting students’ mobile phone dependence should consider psychological capital as an important maintenance and facilitation factor.

### Limitations and future research

Several shortcomings of this study should be acknowledged. First, given the study’s cross-sectional design, the findings do not directly evidence causal relationships. Second, the study did not investigate what participants were using their smartphones for. Four main uses have been identified – academic learning, social networking, online gaming, and entertainment (Jeong et al., 2016) – and different types of use inevitably satisfy different aspects of an individual’s needs. It is unclear to which types of mobile phone use this study’s theoretical model applies. This is an avenue for future research. Third, the low correlation coefficients between physical activity and other psychological indicators may be explained by measuring physical activities in all five areas covered by IPAQ-FL (Occupation, Housework, Transportation, Leisure, and Recreation.) over the course of only 1 week. Moreover, self-reporting may have led to irregularities and non-objectivity. Future studies could use experimental methods to control for such confounding terms to the greatest extent possible. Finally, although the sample size was large (9,569), all participating students attended various universities in Jiangsu Province. Therefore, future studies should recruit participants from a broader geographical area.

## Conclusion

This study revealed a direct negative relationship between physical activity and mobile phone dependence among university students (i.e., higher activity, lower dependence). Moreover, subjective well-being mediated the relationship between physical activity and mobile phone dependence. Psychological capital was also found to play a key role: it moderated the association between subjective well-being and mobile phone dependence, and positively moderated the indirect effect between physical activity and mobile phone dependence *via* subjective well-being (i.e., higher psychological capital, stronger indirect effect).

## Data availability statement

The original contributions presented in the study are included in the article/Supplementary material, further inquiries can be directed to the corresponding author.

## Ethics statement

The studies involving human participants were reviewed and approved by the Ethics Committee of Medical School of Yangzhou University. The patients/participants provided their written informed consent to participate in this study.

## Author contributions

TL, CW, BT, and JY: methodology. TL, CW, HC, and HS: investigation. TL, YJ, HC, and BT: data curation. TL: writing the manuscript. TL, BT, YJ, and JY: project administration. All authors contributed to the article and approved the submitted version.

## Funding

National Office for Philosophy and Social Sciences Funding Number: 22ATY007.

## Conflict of interest

The authors declare that the research was conducted in the absence of any commercial or financial relationships that could be construed as a potential conflict of interest.

## Publisher’s note

All claims expressed in this article are solely those of the authors and do not necessarily represent those of their affiliated organizations, or those of the publisher, the editors and the reviewers. Any product that may be evaluated in this article, or claim that may be made by its manufacturer, is not guaranteed or endorsed by the publisher.
