# Supplementary material for: Relationship between university students’ physical activity and mobile phone dependence: Mediating effect of subjective well-being and moderating effect of psychological capital
Source: Front Psychol. 2022 Dec 29;13:983487. doi: 10.3389/fpsyg.2022.983487 (PMC9835677; doi:10.3389/fpsyg.2022.983487)
Supplement: Supplementary file 1 [file Data_Sheet_1.zip › Code Book.docx]

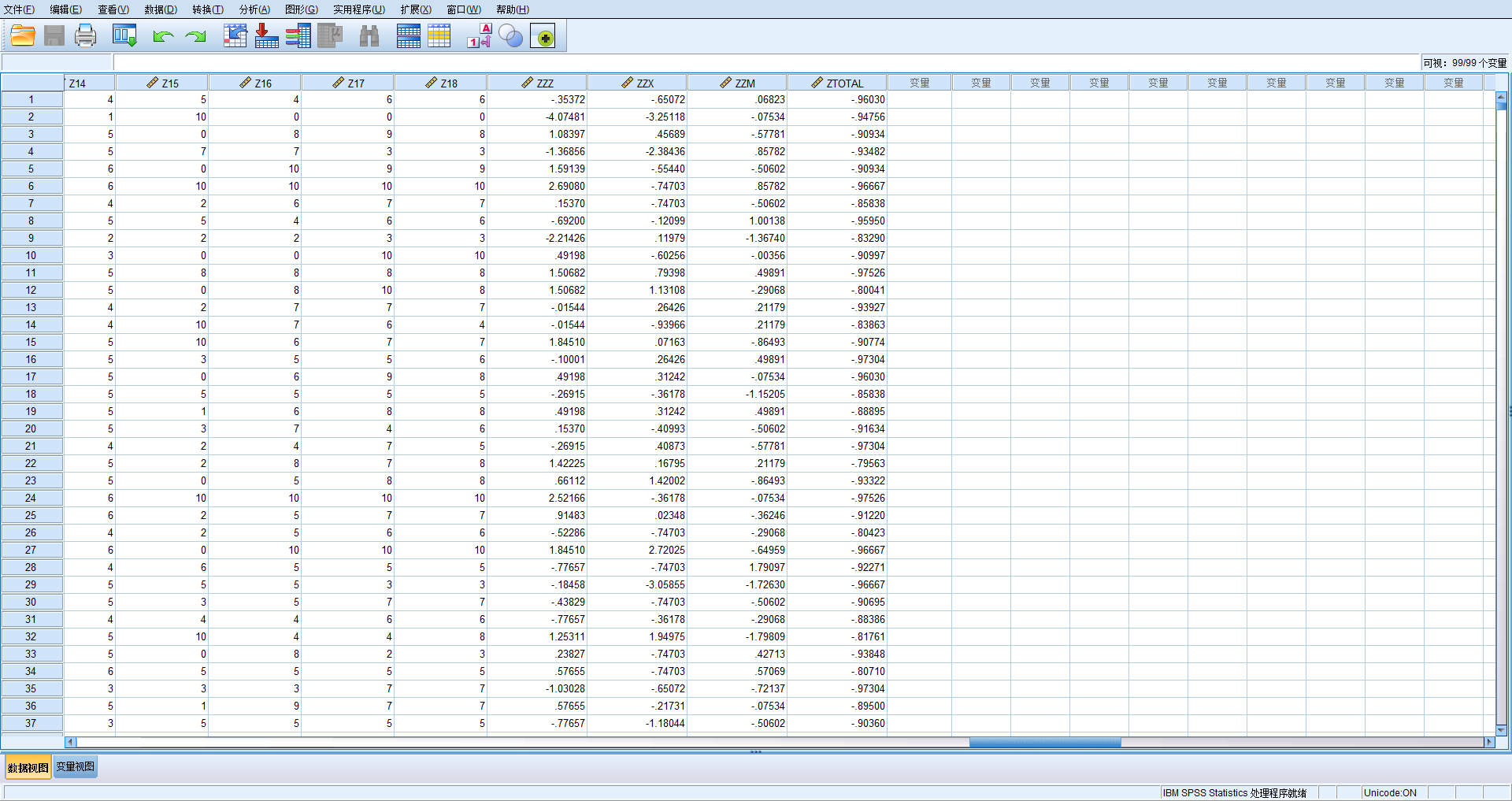
ZTOTAL is the standardized MET value, MET values are calculated from the IPAQ questionnaire


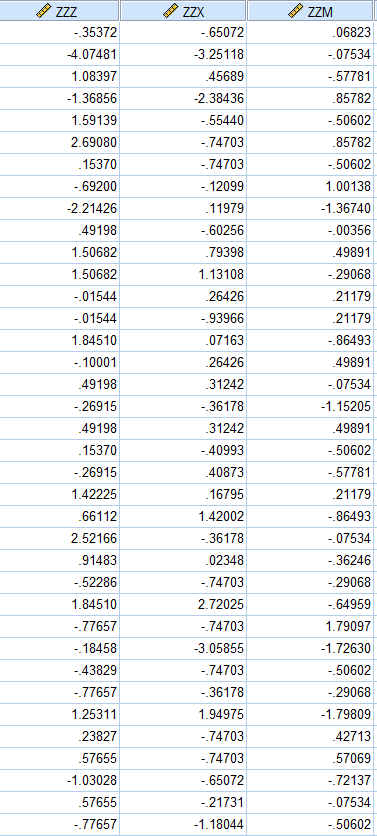


ZZZ, ZZM, ZZX are the sums of the standardized mobile phone dependence questionnaire, subjective well-being questionnaire and psychological capital questionnaire respectively.


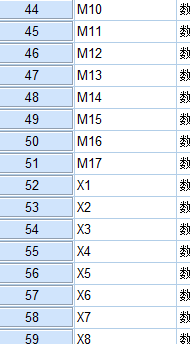


“M”, “X” and “Z” stand for Mobile phone dependency and Subjective well-being.

“M” uses the likert 5-point scoring method.

“1- 5” stand for “Always- Never”.

“X” uses the likert 7-point scoring method.

“1- 7” stand for “Totally Suitable- totally inconsistent”.

“Z” uses the likert 6-point scoring method.

“1- 6” stand for “Excellent- Very bad”.


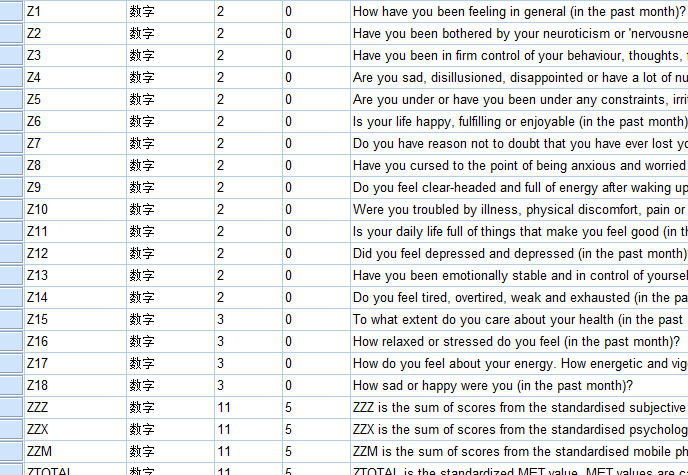


The content of the questionnaire is explained in SPSS and excel sheet.
